# Case Illustrative of the Semi-Decussation of the Optic Nerves

**Published:** 1825-01

**Authors:** Andrew Crawford

**Affiliations:** Physician to the Hampshire County Hospital.


**Art. III.**—*Case illustrative of the Semi-decussation of the Optic Nerves.* By Andrew Crawford, m.d. Physician to the Hampshire County Hospital.

Mrs. B—, aged sixty-five, had a slight hemiplegic attack of the left side, on the 9th of December, 1816. She regained, in a great measure, the use of her limbs; but the following affection of the sight continued from the time of her seizure till her death, about five years afterwards.

When she looked at any object, she could only see one half of it distinctly, the other being very obscure. For example, in looking at a person's face, she could only see distinctly that side of it which was to her right hand. This was equally the case, whether she looked with both eyes or only with the right one; but, when she looked with her left only, the obscurity was greater. When four fingers were held before her, she could see two of them distinctly; the third she could distinguish, but could not see plainly, and the fourth she could not see at all. When she looked at three fingers, she could see two of them pretty plainly,—the first, however, more so than the second; and the third she could not distinguish at all. When she looked at two fingers, she could only see one distinctly.

After she had recovered so as to be able to get out of bed, it was discovered that, although she could only see one half of an object plainly, when held directly before her, yet, if it was moved to her right hand, and she continued to look straight forward, she could see the whole of it distinctly. On the contrary, if it was moved to her left hand, keeping her eyes fixed as before, she could not perceive any of the object at all.

Dr. Wollaston does not notice this last circumstance in the instances he relates; but it appears to me an additional confirmation of his opinion. The defective vision was owing to the insensibility of one-half of the retina of each eye; and that was occasioned, in this instance, probably, by pressure on the right thalamus nervi optici, whence the nervous fibres that proceed (on the supposition of their *semi*-decussation with those from the left thalamus,) finally expand into the right half of the retina of each eye. This part of the retina being insensible, the rays of light which passed to it from objects on the left of the centre of vision, produced no sensation. But, when the eyes were kept fixed, and the object to be looked at moved to the right of the centre of vision, so that all the rays from it passed to the left and sound half of the retina, then the whole of the object became visible.

It should be remarked, that, although the motion of only the *left* side of the body was impaired, yet it was the *right* half of each retina which was insensible.

*Winchester; November* 4, 1824.

